# Metabolomics of Interstitial Fluid, Plasma and Urine in Patients with Arterial Hypertension: New Insights into the Underlying Mechanisms

**DOI:** 10.3390/diagnostics10110936

**Published:** 2020-11-11

**Authors:** Angelika Chachaj, Rafał Matkowski, Gerhard Gröbner, Andrzej Szuba, Ilona Dudka

**Affiliations:** 1 Department of Angiology, Hypertension and Diabetology, Wroclaw Medical University, Borowska 213 Street, 50-556 Wroclaw, Poland; andrzej.szuba@umed.wroc.pl; 2 Department of Oncology, Wroclaw Medical University, 12 Hirszfeld Square, 53-413 Wroclaw, Poland; rafal.matkowski@umed.wroc.pl; 3 Wroclaw Comprehensive Cancer Center, 12 Hirszfeld Square, 53-413 Wroclaw, Poland; 4 Department of Chemistry, Umeå University, Linnaeus väg 6, 901 87 Umeå, Sweden; gerhard.grobner@chem.umu.se (G.G.); ilona.dudka@umu.se (I.D.)

**Keywords:** biomarkers, metabolic phenotyping, 1H NMR spectroscopy, primary hypertension, interstitial fluid, prenodal lymph, lymphatic system

## Abstract

There is growing evidence that lymphatic system plays a pivotal role in the pathogenesis of hypertension. Here, for the first time, the metabolome of interstitial fluid is analyzed in patients with arterial hypertension. Due to ethical issues to obtain human interstitial fluid samples, this study included only oncological patients after axillary lymph node dissection (ALND). These patients were matched into hypertensive (n = 29) and normotensive (n = 35) groups with similar oncological status. Simultaneous evaluation of interstitial fluid, plasma, and urine was obtained by combining high-resolution proton nuclear magnetic resonance (^1^H NMR) spectroscopy with chemometric analysis. Orthogonal partial least squares discriminant analysis (OPLS-DA) provided a clear differentiation between the hypertension and normotensive group, with the discrimination visible in each biofluid. In interstitial fluid nine potential metabolomic biomarkers for hypertension could be identified (creatinine, proline, pyroglutamine, glycine, alanine, 1-methylhistidine, the lysyl group of albumin, threonine, lipids), seven distinct markers in plasma (creatinine, mannose, isobutyrate, glycine, alanine, lactate, acetate, ornithine), and seven respectively in urine (methylmalonate, citrulline, phenylacetylglycine, fumarate, citrate, 1-methylnicotinamide, *trans*-aconitate). Biomarkers in plasma and urine allowed for the identification of specific biochemical pathways involved in hypertension, as previously suggested. Analysis of the interstitial fluid metabolome provided additional biomarkers compared to plasma or urine. Those biomarkers reflected primarily alterations in the metabolism of lipids and amino acids, and indicated increased levels of oxidative stress/inflammation in patients with hypertension.

## 1. Introduction

Arterial hypertension is one of the most prevalent chronic diseases [1] and a leading cause of stroke, heart disease, kidney failure, and premature death [2]. The etiology in 90% cases of hypertension remains unclear and the disease is then classified as “essential” or “primary” hypertension [3]. The pathogenesis of essential hypertension generally depends on the interaction among genetic, environmental, and lifestyle factors. However, the underlying biochemical pathways in hypertension are still not fully understood.

In the pilot study presented here, the main focus was on the evaluation of the metabolome of interstitial fluid to identify metabolic alterations occurring in patients with arterial hypertension; a study complemented also by analyzing the metabolome of plasma and urine in those patients. The application of metabolic phenotyping technologies allowed the profiling of low molecular weight metabolites in these bio-liquids. Those profiles are unique signatures (“fingerprints”), reflecting the metabolic state of patient in response to disease and therapy [4,5]. Metabolites are considered intermediates and end products, resulting from physiological homeostasis, gene expression, and environmental interactions [6]. The application of metabolomics in hypertension studies has the potential to provide identification and novel insights into key pathophysiological processes and related pathways [7,8]. Therefore, this approach can be used to identify metabolites as biomarkers for early diagnosis, disease stage, and efficacy of hypertensive treatment [9,10,11]. Since the therapeutic application of altered metabolites may affect phenotypes [12,13,14,15,16], the evaluation of metabolomic profiling of individual patients can be used to design personalized hypertensive treatment in the future.

Previous metabolomic studies on hypertension have been mainly based on—easy to obtain—blood and urine samples [7,17,18,19,20,21]. However, the metabolomics of blood and/or urine might not at all fully reflect tissue-level changes associated with hypertension [22]. Therefore, other bioliquids gained growing interest recently such as from skin interstitium and the lymphatic system. Both seem to play pivotal roles in the pathogenesis of cardiovascular disorders, including hypertension [23,24,25,26,27]. The lymphatic system governs the transport of interstitial fluids from extracellular space to the blood circulation, thus, maintaining peripheral tissue homeostasis, including fluid and lipids balance throughout the body. The lymphatic vessels drain products of tissue metabolism and catabolism, as well as circulating immune cells and transport them to the regional lymph nodes [28]. Metabolomics of lymph has never been evaluated before in arterial hypertension. Since human lymph samples cannot be obtained easily due to ethical reasons, only oncological patients after axillary lymph nodes dissection (ALND) were included in the study. For cancer patients, arterial hypertension is the most common co-morbidity; a fact which can significantly influence cancer care and clinical outcomes. Therefore, it is important to diagnose and manage hypertension in this group of cancer patients [29].

The samples of interstitial fluid in our study were obtained via drains placed in the axillary area after ALND. Interstitial fluid has the same composition as prenodal lymph, since modification of the lymph composition occurs first in the lymph nodes [28]. Most of the collected fluid was prenodal lymph from the entire upper limb on the operated side, since lymph could not be transferred through the lymphatic vessels within the armpit due to the damage to the lymphatic vessels during ALND. A minor fraction of samples from the drains constituted the inflammatory fluid from the operated armpit.

In this study, we used not only interstitial fluid samples (to our knowledge, for the first time in the metabolomic study in hypertension), but also plasma and urine samples from hypertensive and normotensive (as reference) oncological patients. Simultaneous metabolomics profiling of these three biofluids was carried out using high-resolution proton nuclear magnetic resonance (^1^H NMR) spectroscopy, a widely used analytical techniques to analyze biofluid metabolites [30]. The main advantages of NMR based metabolomics approach are the robustness of the technique, the unambiguous identification of individual metabolites and their quantification, and the non-destructiveness to the samples [30]. Thus, NMR spectroscopy, although less sensitive than mass spectrometry (MS), allows for the acquisition of a large amount of high-quality reproducible data [31]. Combining NMR-derived metabolomic data from three biofluids enabled us to identify pathophysiological pathways in arterial hypertension and the scale of occurring biochemical alterations.

## 2. Materials and Methods

### 2.1. Patient Cohort 

This study enrolled 64 patients of the Wroclaw Comprehensive Cancer Center in Poland who had undergone ALND. All patients had cancer according to histopathological examination, and 29 of them had arterial hypertension, while the other 35 were normotensive and acted as the reference group. Study groups were matched at the stage of the patient recruitment to have the same oncological status. The majority were diagnosed with breast cancer (i.e., 25 patients in the hypertensive cancer group and 29 patients in the normotensive cancer group). Exclusion criteria were secondary hypertension, diabetes mellitus, and kidney failure. Diagnosis of hypertension consisted of clinical and family history, physical examination, body mass index (BMI), and at least two blood pressure measurements by Riva Rocci method and routine laboratory investigations (i.e., complete blood count, electrolytes, glucose, creatinine, and urinalysis). All patients in the hypertensive group were treated by orally administered antihypertensive therapy with beta-blockers (BBs) or a combination of 2 to 4 agents including diuretics, BBs, angiotensin-converting-enzyme inhibitors (ACEIs), angiotensin II receptor blockers (ARBs), and calcium channel blockers (CCBs). 

Ethical approval was obtained from the Ethics Committee of Wroclaw Medical University, Poland (KB-40/2011). Written informed consent was obtained from each participant following the principles outlined in the Declaration of Helsinki.

### 2.2. Sample Collection

Fasting blood samples and urine samples were taken in the morning before the ALND was carried out. Peripheral blood samples were collected by venipuncture of the antecubital vein using the Sarstedt S-Monovette system (Sarstedt AG & Co., Nümbrecht, Germany). Then, blood samples were centrifuged at 3000× *g* for 10 min at 4 °C to isolate plasma. Interstitial fluid samples were taken via drains in the armpit after surgery. Collection of interstitial fluid samples occurred 3–4 days after ALND to obtain clear interstitial fluid without admixture of blood. From each individual three biofluids (interstitial fluid, plasma, and urine) were taken and stored until analysis. One urine sample was missing from a patient in the normotensive group.

### 2.3. ^1^H NMR Analysis of Plasma, Lymph and Urine

Preparation of the plasma and urine samples for metabolomic analysis was carried out according to Dona et al. [31]. For interstitial fluid samples, preparation protocol of plasma was applied. Briefly, before analysis, plasma and interstitial fluid samples were thawed and centrifuged at 13,000× *g* for 10 min at 4 °C to remove insoluble material. Then, 300 μL of plasma/interstitial fluid were mixed with 300 μL of 1.5 M of deuterated phosphate buffer (NaH_2_PO_4_ and K_2_HPO_4_, including 0.1% TSP, pH 7.47) and transferred into 96-well plates for NMR spectroscopy using a Gilson robot. Urine samples were thawed and centrifuged at 12,000× *g* for 5 min at 4 °C and then 540 μL of the sample was mixed with 60 μL of phosphate buffer pH 7.4 containing 0.1% TSP, pH 7.47 by Gilson robot. Quality control (QC) samples were prepared for each biofluid by pooling all samples to monitor the analytical variability of the metabolic profiling platform. 

^1^H NMR spectra were acquired at 311 K for plasma and interstitial fluid samples and 301 K for urine samples on a Bruker 600 MHz AVANCE III (Rheinstetten, Germany) spectrometer equipped with a 5 mm BBO broadband (1H/19F/2D) z-gradient cryo-probe. Setup of experiments for plasma and urine samples was carried out, as described previously [31]. Taking into account the similarity between plasma and interstitial fluid, we applied identical conditions for both. For each plasma and interstitial fluid sample, two 1D NMR experiments were acquired with the first being a standard ^1^H NMR spectrum (NOESY) with water suppression, a 90° flip angle, a cycle delay of 4 s, an 18-kHz spectral width, 98,304 data points, an 0.01 s mixing time, and a total 32 scans after 4 dummy scans. The second experiment was a T_2_ edited ^1^H CMPC (Car-Purcell-Meiboom-Gill) NMR spectrum (with water suppression for enhanced visualization of compounds with low molecular weight. By analyzing NOESY NMR spectra of plasma and interstitial fluid, we detected very similar metabolomic profiles with respect to lipids and lipoproteins. Therefore, we applied the same parameters for CPMG NMR experiments for interstitial fluid as before for plasma. As checked during setting up experiments those parameters were suitable for a comprehensive analysis of both biofluids. The corresponding NMR spectrum was acquired with a recycle delay of 4 s, 12-kHz spectral width, 73,728 data points, 30 ms total spin-echo time, total of 64 scans, and 4 dummy scans. For urine samples, primary acquisitions were made using a standard 1-D pulse program (recycle delay (RD)-90°-t1-90°-tm-90°-acquire free induction decay (FID)). The 90° pulse length was adjusted to ∼12 μs. A total of 64 scans were recorded into 32 K data points with a spectral width of 20 ppm. An exponential function was applied to the FID before the Fourier transformation, which resulted in a line broadening of 0.3 Hz. 

All acquired NMR spectra were manually corrected for phase and the baseline using TopSpin 2.1 (Bruker Biospin, Rheinstetten, Germany). Subsequently, NMR spectra were aligned using icoshift 1.2 and manual integration of peaks was performed to a linear baseline on all spectra in parallel by using an in-house developed Matlab routine, as described previously [32]. The integrated data from plasma/interstitial fluid were normalized to the total sum of the spectrum to give the same total integration value for each spectrum. Urine data were normalized using Probabilistic Quotient Normalization (PQN). Metabolite identification was carried out using Chenomx NMR suite professional (version 7.72, Chenomx, Inc., Edmonton, AB, Canada).

### 2.4. Multivariate and Statistical Analyses

Normalized NMR data sets were unit variance (UV) scaled before multivariate analysis. Multivariate data analysis methods, principal component analysis (PCA) and orthogonal partial least squares discriminant analysis (OPLS-DA) were used to reduce the dimensionality and to enable the visualization of the separation of the study groups (SIMCA 14.0, Umetrics, Umeå, Sweden). All OPLS-DA models used 7-fold cross-validation to assess the predictive ability of the model (Q2). Further validation of the models was carried out by using cross-validation ANOVA (CV-ANOVA). Important metabolites differentiating study groups were selected based on loadings plots (|*p*| > 0.10) from OPLS-DA models and results of the univariate analysis using the Student’s *t*-test. The Student’s *t*-test was selected, based on the outcome of the Shapiro-Wilk test for normality. *p*-Values of less than 0.05 were considered as statistically significant. To avoid the influence of potential confounders, each selected metabolite was further confirmed by adjusting for age and the body mass index (BMI), using linear regression models based on the MATLAB command *fitlm*.

## 3. Results

### 3.1. Characteristics of the Study Subjects

Basic demographic and clinical data of the study groups are summarized in Table 1. There were no significant differences in gender between two groups based on statistical analysis (*p* > 0.05). Men in both study groups constituted about 10%. However, age and body mass index (BMI) were significantly different between both study groups. 

### 3.2. Metabolic Profiling of ^1^H NMR Spectra of Plasma, Interstitial Fluid and Urine 

**Figure 1:**
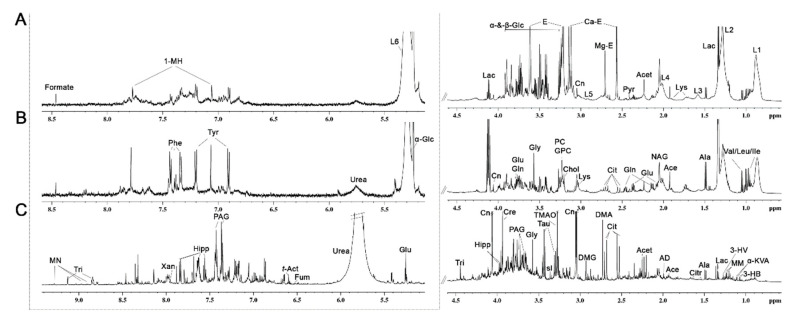
Representative 600-MHz high-resolution proton nuclear magnetic resonance (^1^H NMR) spectra of plasma (**A**), interstitial fluid (**B**) and urine (**C**). The regions of δ 5.0–8.5 for plasma and interstitial fluid and δ 5.0–9.4 for urine were magnified compared with the corresponding regions δ 0.6–4.5 for purposes of clarity. Abbreviations: L1: lipid-CH_3_; L2: lipid –(CH_2_)n; Lac: lactate; L3: lipid CH_2_-CH_2_=O; Lys: lysine; L4: lipid CH_2_-CH=; Ace: acetate; Pyr: pyruvate; Ca-E: Ca–EDTA; Mg-E: Mg–EDTA;L5: lipid =CHCH_2_-CH=; Cn: creatinine; E: EDTA; α-&-β-Glc: α-&-β-glucose; L6: lipid –CH=CH-; 1-MH:1-methylhistidine; Val: valine; Leu: leucine; Ile: isoleucine; Ala: alanine; Acet: acetone; NAG: N-acetyl glycoprotein; Glu: glutamate; Gln: glutamine; Cit: citrate; Chol: choline; GPC: glycerophosphocholine; PC: phosphocholine; Gly: glycine; α-Glc: α-glucose; Tyr: tyrosine; Phe: phenylalanine; 3-HB: 3-hydroxybutyrate; α-KVA: α-ketoisovaleric acid; MM: methylmalonate; 3-HV: 3-hydroxyisovalerate; Citr: citrulline; AD: acetamide; DMA: dimethylamine; DMG: dimethylglycine; TMAO: trimethylamine-N-oxide; Tau: taurine; sI: scyllo-inositol; PAG: phenylacetylglycine; Cre: creatine; Hipp: hippurate; Tri: trigonelline; Fum: fumarate; *t*-Act: *trans*-aconitate; Xan: xanthine; MN: 1-methylnicotinamide.

Untargeted metabolomics analysis was carried out using the ^1^H NMR spectra of all bio-liquid samples. R Figure 1A–C, show representative 600 MHz ^1^H CPMG NMR spectra of plasma and interstitial fluid and ^1^H NMR NOESY 1D spectrum of urine from the normotensive group. Inspection of NMR spectra of plasma and interstitial fluid reveals a wide variety of metabolite resonances, mainly including BCAA, alanine, lactate, lysine, acetate, N-acetyl glycoprotein (NAG), glutamate, glutamine, pyruvate, citrate, creatinine, glycerophosphocholine, phosphocholine, glycine, urea, tyrosine, phenylalanine, 1-methylhistidine, formate, and lipid species. Several metabolites were also identified in urine, mainly including 3-hydroxybutyrate, α-ketoisovaleric acid, methylmalonate, 3-hydroxyisovalerate, citrulline, acetamide, dimethylamine, dimethylglycine, trimethylamine-N-oxide, taurine, scyllo-inositol, phenylacetylglycine, creatine, hippurate, trigonelline, fumarate, *trans*-aconitate, xanthine, and 1-methylnicotinamide.

### 3.3. Biomarker Identification for Hypertension

**Figure 2:**
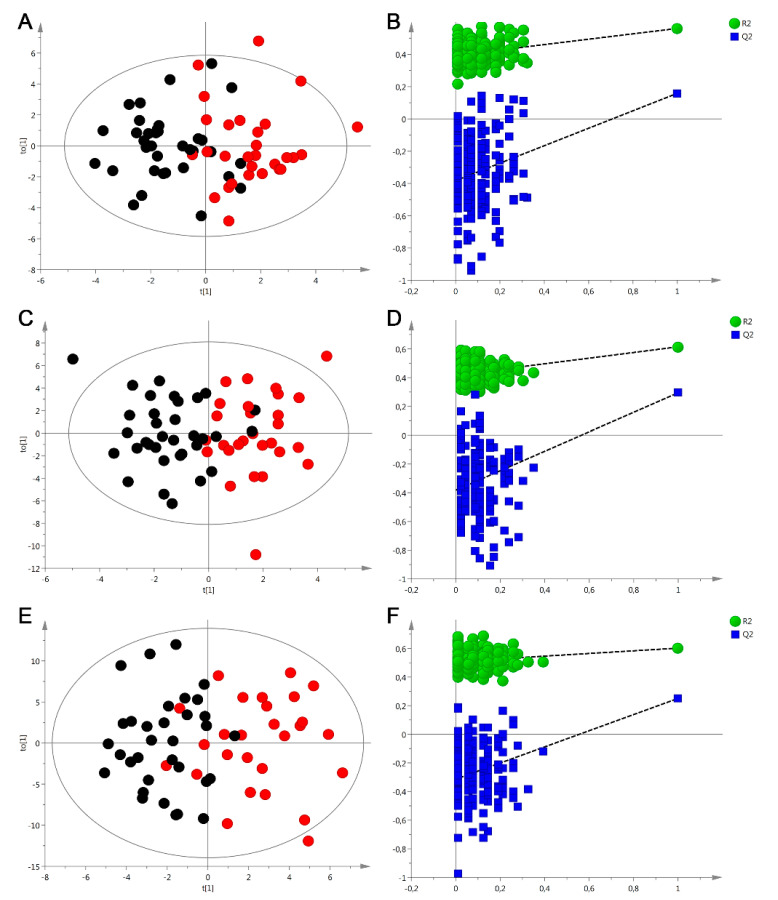
Multivariate analysis of metabolomics data from hypertensive patients (red dots) and normotensive patients (black dots). (**A**) Orthogonal partial least squares discriminant analysis (OPLS-DA) score plot of plasma ^1^H NMR data; (**B**) plot obtained after performing random permutation test with 200 permutations on OPLS-DA model of plasma data; (**C**) OPLS-DA score plot of interstitial fluid^1^H NMR data; (**D**) plot obtained after performing random permutation test with 200 permutations on OPLS-DA model of interstitial fluid data; (**E**) OPLS-DA score plot of urine ^1^H NMR data; (**F**) plot obtained after performing random permutation test with 200 permutations on OPLS-DA model of urine data.

To probe the metabolic variations, analysis of all data for the three bio-liquids was performed by using SIMCA software, which provides discrimination and significant variables selection. First, an unsupervised PCA analysis was applied to identify potential outliers (one plasma sample, three interstitial fluid samples, and three urine samples), and exclude them from subsequent modeling processes. OPLS-DA analysis for each data set allowed a maximum on sample group separation and identification of the discriminating metabolites. The relevant OPLS-DA score plots of plasma, interstitial fluid, and urine are shown in Figure 2A,C,E, respectively. For all bio-fluids, good separations were found between hypertensive subjects and normotensive subjects, with differences being visible in plasma, interstitial fluid, and urine metabolic profiles between both study groups. Goodness of fit values and predictive ability values (explained variance R2Y and predicted variance Q2) indicated that all models possessed a reasonable fit and predictive power: for plasma data: R2Y = 0.727, Q2 = 0.275; for interstitial fluid data: R2Y = 0.605, Q2 = 0.242; and for urine data: R2Y = 0.602, Q2 = 0.251). A CV-ANOVA test showed highly significant variation related to the separation of groups: for plasma data: *p*-value = 0.005; for interstitial fluid data: *p*-value = 0.003; and for urine data: *p*-value = 0.003. Cross-validation analysis using 200 random permutations are shown separately for plasma data in Figure 2B, interstitial fluid data in Figure 2D, and for urine data in Figure 2F. The R^2^ and Q^2^ intercepts values determined after permutations were: for plasma data: 0.52 and −0.56, respectively; for interstitial fluid data 0.42 and −0.39, respectively and for urine data 0.51 and −0.33, respectively. Presented validation plots confirmed the robustness of the OPLS-DA models for plasma and interstitial fluid data. Consideration was taken in evaluating plot for urine data, as all of the Q2 (cum), but not all R2 (cum) values were lower than the original values in the validation plot. However, taking into account results of CV-ANOVA analysis, the model for urine data is robust and valid. 

**Figure 3:**
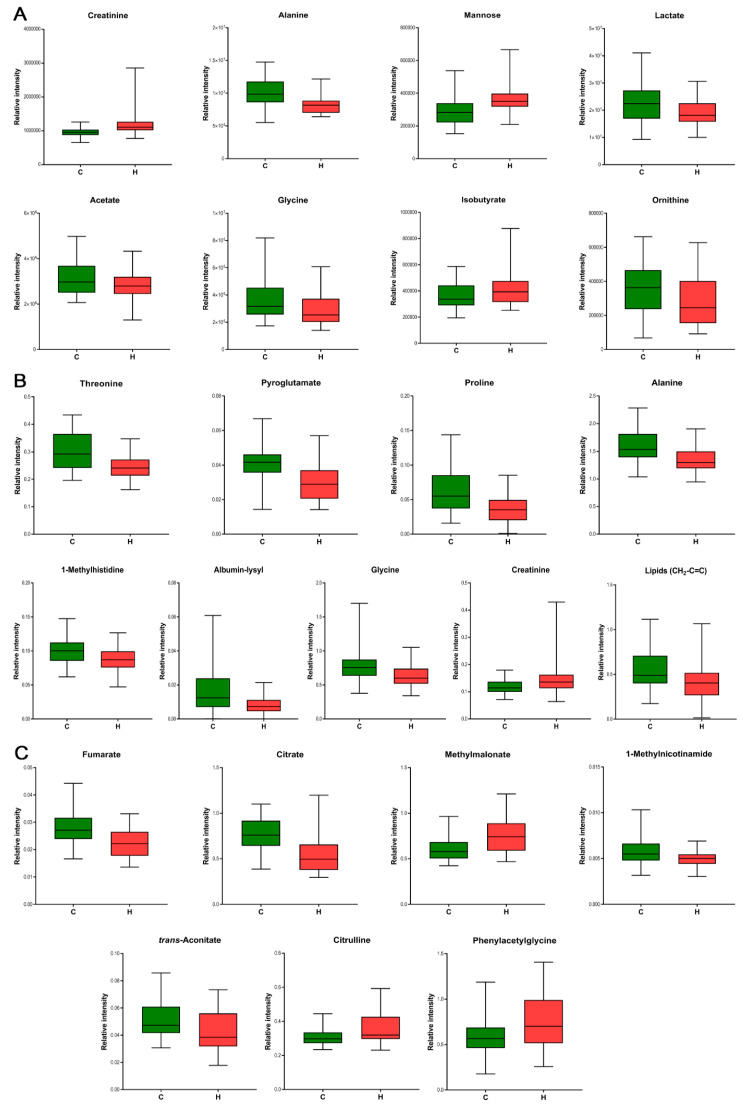
Significantly altered metabolites in the hypertensive group (red box plot) compared to the normotensive group (green box plot) in plasma (**A**), interstitial fluid (**B**), and urine (**C**).

**Figure 4:**
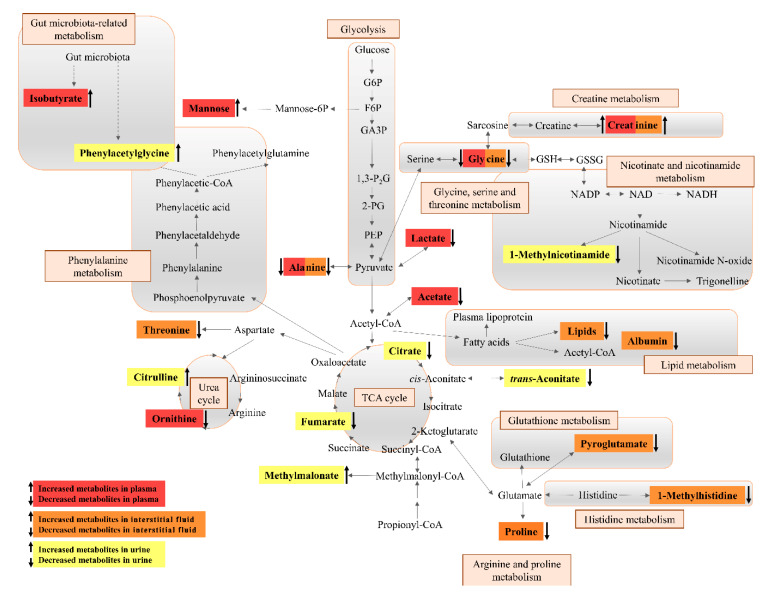
Simplified representation of metabolomic pathways, showing significantly altered metabolites in hypertensive patients compared to normotensive patients in plasma (red), interstitial fluid (orange), and urine (yellow).

Potential metabolic biomarkers of hypertension in patients were selected based on OPLS-DA loading plots (|*p*| > 0.10) and the results of the *t*-test (*p* < 0.05). By applying this criterion as previously described [33], metabolites were identified whose distribution patterns were important for class separation. This way, 21 significant metabolites were identified (see Table 2). Adjustment for age and BMI by linear regression for each selected metabolite revealed that most of them were independent of these covariates. The plasma of hypertensive patients, contained eight metabolites with significant changes in concentrations compared to normotensive patients, with three metabolites increased and five decreased (Figure 3A). The metabolite profile of interstitial fluid in the hypertensive group showed a strong increase in one metabolite and a decrease in eight metabolites (Figure 3B). Three of them were common for plasma and interstitial fluid and had the same pattern of changes in hypertensive patients. The hypertensive group showed in urine an increase of three metabolites and a decrease of four metabolites (see also Figure 3C). As the group here consists of solely oncological patients, with most of them having breast cancer, any relation between cancer type and metabolic profile were also studied. However, the significant metabolites used for differentiation and their relative levels were typical for all hypertensive patients. Therefore, we could develop a simplified representation of the biochemical pathways network (see Figure 4), which reflects the metabolic variations in patients with arterial hypertension. This network is based on all indicative metabolomic biomarkers of hypertension which were identified in the interstitial fluid, blood, and urine biofluids. This map clearly reflects the important interactions between the altered metabolic pathways in plasma, interstitial fluid, and urine in patients with arterial hypertension. 

## 4. Discussion

An increasing number of cancer patients with pre-existing cardiovascular diseases represents a severe challenge for clinicians and a new frontier for intense research towards better diagnosis and therapy. In this pilot study, multivariate analysis of ^1^H NMR derived metabolic profiles of interstitial fluid, plasma, and urinary samples successfully identified the metabolomic differences between oncological patients with and without hypertension. Based on our knowledge, research of arterial hypertension in cancer patients with application of metabolomics on interstitial fluid, has never been studied before. By using complimentary metabolomics profiling of plasma and urine on those patients here, our findings could be compared to previous plasma and urine based metabolomic studies on non-cancer hypertensive patients. The metabolomics findings for interstitial fluid here are unique in the context of hypertension research. Through analyzing the metabolomic patterns present in different body fluids of the same individual we obtained a deeper understanding of the metabolic pathways altered in the setting of treated hypertension in oncological patients (Figure 4).

The metabolomic profiles of each biofluid alone already provided a clear and strict differentiation between the hypertensive and the normotensive group. To our knowledge, the study here is the first one showing that the metabolomic analysis of interstitial fluid enables a clear differentiation between both groups and provides information to understand the biochemical pathways involved in arterial hypertension. It complements metabolomics studies based solely on plasma and urine biofluids. 

### 4.1. Differences in Metabolite Levels in the Hypertensive Group vs. Normotensive Group

#### 4.1.1. Interstitial Fluid

The increased levels of creatinine and decreased levels of amino acids in interstitial fluid of the hypertensive patients were in line with the results of the metabolic profiles found in the plasma of those patients. A higher concentration of pyroglutamate in interstitial fluid in this patient group might indicate alterations in biochemical pathways associated with glucose metabolism [34,35]. Proline and threonine are decreased in interstitial fluid in the hypertensive group; an observation revealing complex alteration of amino acids metabolism in hypertension [36]. However, their decreased concentrations in interstitial fluid altogether with 1-methylhistidine, glycine, and albumin lysyl may also indicate increased oxidative stress and inflammation associated with hypertension [7,37]. Proline participates in redox reactions [38], and has the potential to scavenge free radicals in vitro [39]. Threonine itself is involved in many physiological processes, including various immune functions [40,41]. The 1-methylhistidine can be metabolized into anserine, which is an important free radical scavenger [42]. Glycine possesses also anti-inflammatory, immunomodulatory, antioxidant, and cytoprotective properties [43,44]. Therefore, it acts against oxidative stress under various pathological situations, e.g., as a cardioprotective agent in coronary arterial endothelial cell inflammation [45], and it protects against oxidative damage in patients with metabolic syndrome [15]. Although there are no reports about lysyl groups of albumin involved in hypertension, its lower level in interstitial fluid in our hypertensive group may also indicate a higher degree of oxidative stress associated with hypertension, as previous findings have suggested [46].

Remarkably, potential biomarkers of oxidative stress and inflammation were much more clearly visible in interstitial fluid compared to plasma and urine of hypertensive patients. This is in line with a recent proteomic study [47], in which the interstitial fluid was found to be specifically enriched with mediators of energy/redox metabolism. Oxidative stress has gained attention as one of the fundamental mechanisms involved in the development of hypertension [48], with increased ROS production in patients with various types of hypertension [49]. Our results obtained for interstitial fluid samples of hypertensive patients provide further evidence that increased oxidative stress is involved in the pathogenesis of hypertension.

Decreased concentrations of lipids and threonine in interstitial fluid in those patients are, in turn, consistent with known disruptions in lipid metabolism in hypertension [19,50]. 

Lipids are absorbed from peripheral tissues to the lymphatic capillaries in the process of reverse cholesterol transport (RCT). Reduced levels of fatty acids in the interstitial fluid presumably indicate a reduction of lipid transport from peripheral tissues to the circulatory system, i.e., decreased RCT [51]. This decrease might support atherosclerosis development [52,53]. Elevated threonine levels were reported to be inversely associated with a reduced risk of atherogenic lipid profile, including decreased levels of small dense low-density lipoprotein cholesterol (LDL), remnant-like particle cholesterol and triglycerides [54]. However, threonine may be also decreased as a consequence of the intake of beta-blockers and thiazide diuretics [55]. Some of our hypertensive patients took these medicines, thus, the effect of drugs on the concentration of this amino acid cannot be ruled out completely. 

Decreased levels of glycine in interstitial fluid and plasma, and proline in interstitial fluid may also be associated with a dysregulation in elastin and collagen synthesis associated with hypertension. Glycine is one of the main components of collagen and its role in maintaining collagen structure is critical [56,57]. It has been shown that lower levels of glycine are associated with impaired collagen and elastin formation in hypertension, causing a reduced elasticity of the arterial wall [58,59,60]. Proline is another metabolite, the conversion of which into 4-hydroxyproline plays an important role in collagen stability [61]. However, proline may be also converted to ornithine or L-arginine. Thus, decreased levels of proline in interstitial fluid in the cancer hypertension group may reflect alterations in the arginine/NO pathway [62].

#### 4.1.2. Plasma

The plasma metabolomic profile revealed an increased concentration of creatinine in the hypertensive group. Creatinine is a widely used biomarker for the evaluation of renal function in clinical practice [63]. Elevated creatinine concentrations indicate an increased risk of cardiovascular consequences of hypertension [64,65].

In the hypertensive group decreased levels of plasma amino acids were found, including for ornithine, glycine, and alanine. Changes in amino acids composition are seen as a potential biomarkers of hypertension [36,66]. Amino acids are the basic units for protein synthesis, and affect such functions as proliferation, immune response, and the regulation of tricarboxylic acid cycle (TCA cycle) [66]. Ornithine is a degradation product of arginine with alterations in the arginine metabolic pathways, causing reduced nitric oxide (NO) bioactivity; a prominent feature for endothelial dysfunction in hypertension [67]. In agreement with our results, the glycine level was reported to be lowered in patients with arterial hypertension [9,20,36]. A lower risk of coronary heart disease seems to be associated with higher glycine concentrations and reduced blood pressure [68,69]. Indeed, glycine supplementation has been shown to lower blood pressure in rodents [13,14] and in humans [15], but even in rat models for metabolic syndrome [70]. In contrast, recent reports on the significance of alanine levels in hypertension were conflicting, with downregulated alanine levels [21,66] and others upregulated ones [18,71,72] being reported. 

Our hypertensive group showed also increased level of mannose in the plasma. Plasma mannose levels have been tightly associated with atherogenesis [73], cardiovascular diseases [74,75], and mortality [76], and indirectly also with markers of inflammation (including CRP), creatinine, lower glomerular filtration rate, and with urine albumin excretion [74].

Here, we also observed decreased levels of lactate in the hypertensive group. Lactate is formed under hypoxic conditions, and it appears in serum during ischemia caused by e.g., insufficient blood flow due to atherosclerosis [77]. In our study, decreased plasma lactate levels might be caused by pyruvate impairment in hypertension, associated with amino acids alterations. However, some reports indicate higher lactate levels related to higher blood pressure [78,79]. Our hypertensive patients were under treatment with antihypertensive drugs, and had normal blood pressure, hence, the lactate level was not increased in this group.

We further observed that the hypertension group had increased isobutyrate and decreased acetate plasma concentrations. These alterations may reflect novel physiological connections of hypertension with the functioning of the gut microbiota [7,17,18,80]. Compared to healthy controls, hypertension was associated with decreased microbial richness and diversity, and the microbiome characteristic in the pre-hypertension group was quite similar to that found in the hypertension group [7]. Furthermore, fecal transplantation from hypertensive human donors to germ-free mice [7], and from hypertensive rats to normotensive rats [81], resulted in elevated blood pressure in the hosts. 

#### 4.1.3. Urine

Hypertensive patients had a significantly lower urinary concentration of 1-methylnicotinamide (1-MN) in comparison to the normotensive group. 1-MN is a major metabolite of nicotinamide and exerts antithrombotic and anti-inflammatory effects through its direct action on the endothelium [82,83]. Chronic treatment of diabetic or hypertriglyceridemic rats with 1-MN had the potential to reverse the impairment of NO-dependent endothelial dysfunction [16].

The increased concentration of citrulline in urine in the hypertensive group may be recognized as the biomarker of alterations occurring in the urea cycle [84]. However, urea cycle enzymes are highly regulated by a wide range of hormones, pro- and anti-inflammatory cytokines, and other agents [84]. In turn, the TCA cycle is central in the regulation of energy and cell metabolism, and may be significantly altered in resistant hypertension [85]. Our results concerning TCA cycle intermediates (i.e., decreased concentrations of citrate and fumarate) and substrates for TCA cycle intermediates (i.e., decreased concentration of *trans*-aconitate and increased level of methylmalonate—they are converted into *cis*-aconitate and succinate, respectively) indicate impaired TCA cycle flux in our hypertensive group in comparison to the normotensive one, which is in agreement with an earlier study [86].

Changes in metabolites associated with gut microflora present in plasma of hypertensive patients were also visible in their urine, as increased concentrations of phenylacetylglycine. This relationship between urine metabolites related to host-gut microbial pathways and blood pressure has been described recently [17,87]. 

### 4.2. Limitations

We are aware of some limitations of our study. First, this pilot study consists of a limited number of patients after ALND. Nevertheless, the selection of this specific group of patients enabled us to collect very unique interstitial fluid samples. The interstitial fluid has never been evaluated in the context of metabolomics in arterial hypertension, despite various previous studies indicating a potential role of the lymphatic system in the pathophysiology of hypertension [24,25]. Additionally, our samples of interstitial fluid, which mostly came from disrupted lymphatic vessels (prenodal lymph), contained minor fractions of inflammatory interstitial fluid originating from the operated armpit. However, both groups of patients underwent the same type of surgery, performed by the same surgical team. Therefore, this admixture should not have any severe impact on the robustness of the results. Second, the hypertensive group was treated with different antihypertensive drugs; a treatment which can affect the plasma metabolomic profiles of patients with essential hypertension [10,88]. Therefore, the results presented here cannot rule out an impact of the used drugs on the metabolomic changes seen. In a previous study, we found that the intake of beta-blockers and diuretics could decrease threonine levels [55]. Third, our patient groups differed in age and BMI, which are known significant factors that influence metabolic profiling. However, we are confident that the applied adjustment for age and BMI enabled a faithful comparison of both groups, with most metabolites found to be independent of these covariates. 

## 5. Conclusions

The management of hypertension plays an important role in the treatment of oncological patients. Our pilot study demonstrates that metabolomics of interstitial fluid can clearly differentiate the cancer hypertensive group from the cancer normotensive one. Here, we could show that interstitial fluid, which has not been explored in hypertension studies previously, was unique in identifying distortions in the lipid metabolism; distortions that were not observable at all in plasma or urine. The impact of oxidative stress/inflammation and alterations in mono-amino acids metabolism were visible in plasma samples, but were much more pronounced in the metabolic profiles originating from interstitial fluid. Thus, interstitial fluid and plasma exhibit both common and distinct metabolic patterns, revealing their independent and synergistic biological implications. 

The metabolites identified in our study are not only potential valuable biomarkers of hypertension in oncological patients, but also provide molecular information about underlying biochemical mechanisms; information that will be important for designing novel specific diagnostic and tailored therapeutic approaches. 

## Figures and Tables

**Table 1:** Demographic and medical data on hypertensive vs. normotensive patients.

Clinical Data	Hypertensive Group (n = 29)	Normotensive Group (n = 35)	*p*-Value
Age, y	65.8 ± 8.4	53.4 ± 12.1	<0.0001
BMI, kg/m^2^	29.5 ± 4.9	26.3 ± 4.3	0.007
Gender, % male	13.8	11.4	
Duration of hypertension, y	11.5 ± 10.2		
SBP, mm Hg	127.1 ± 10.9		
DBP, mm Hg	80.4 ± 6.1		
Antihypertensive drugs, in total	1.8 ± 0.7		
ACEi, %	48.3		
ARB, %	17.2		
Diuretic, %	34.5		
Calcium channel blocker, %	27.6		
β-Blocker agent, %	37.9		
Clonidine, %	3.3		
Familiar history of hypertension, % yes	82.8		
Cancer type:			
Breast cancer	25	29	
Cutaneous melanoma	3	4	
Axillary tumor	1	2	

Values are expressed as percentage (%) or mean ± SD. Abbreviations: ACEi: angiotensin converting enzyme inhibitor; ARB: angiotensin receptor blocker; BMI: body mass index; DBP: diastolic blood pressure; SBP: systolic blood pressure.

**Table 2:** Significant metabolites differentiating hypertensive group from normotensive group in plasma, interstitial fluid, and urine.

Metabolite	Matrix	p[1]	*p*-Value (*t*-Test)	*p*-Value Adjusted for Age	*p*-Value Adjusted for BMI	Pathway	Superpathway
Mannose	Plasma	0.24	2.36 × 10^−3^	7.18 × 10^−2^	7.34 × 10^−3^	Fructose, Mannose and Galactose Metabolism	Carbohydrate
Lactate	Plasma	−0.20	1.33 × 10^−2^	6.07 × 10^−3^	3.92 × 10^−3^	Glycolysis, Gluconeogenesis, and Pyruvate Metabolism	Carbohydrate
Isobutyrate	Plasma	0.18	3.56 × 10^−2^	2.54 × 10^−2^	2.85 × 10^−2^		Gut microbiota
Acetate	Plasma	−0.13	2.45 × 10^−2^	2.15 × 10^−2^	1.26 × 10^−1^		Gut microbiota
Ornithine	Plasma	−0.19	4.21 × 10^−2^	9.76 × 10^−2^	1.01 × 10^−2^	Urea cycle; Arginine and Proline Metabolism	Amino acid
Creatinine	Plasma Lymph	0.27 0.19	4.36 × 10^−3^ 3.86× 10^−2^	3.97 × 10^−3^ 3.31 × 10^−2^	9.34 × 10^−3^ 1.37 × 10^−1^	Creatine metabolism	Amino acid
Alanine	Plasma Lymph	−0.29 −0.30	2.98 × 10^−4^ 2.15× 10^−3^	1.36 × 10^−3^ 3.10 × 10^−3^	7.43 × 10^−3^ 6.79 × 10^−3^	Alanine and aspartate metabolism	Amino acid
Glycine	Plasma Lymph	−0.21 −0.25	2.82 × 10^−2^ 8.96× 10^−3^	5.39 × 10^−1^ 2.96 × 10^−1^	3.51 × 10^−1^ 7.04 × 10^−2^	Glycine, serine and threonine metabolism	Amino acid
Threonine	Lymph	−0.27	2.21× 10^−4^	5.71 × 10^−3^	3.64 × 10^−4^	Glycine, Serine and Threonine Metabolism	Amino acid
Pyroglutamate	Lymph	−0.27	3.72× 10^−4^	6.54 × 10^−3^	3.07 × 10^−3^	Glutathione metabolism	Amino acid
Proline	Lymph	−0.26	1.22 × 10^−3^	2.73 × 10^−2^	3.99 × 10^−3^	Urea cycle; Arginine and Proline Metabolism	Amino acid
1-Methylhistidine	Lymph	−0.18	2.54× 10^−3^	2.43 × 10^−2^	2.35 × 10^−3^	Histidine metabolism	Amino acid
Albumin-lysyl	Lymph	−0.20	3.42× 10^−3^	8.77 × 10^−2^	1.72 × 10^−2^	Protein	Lipid
Lipids (CH_2_-C=C)	Lymph	−0.16	4.79× 10^−2^	3.73 × 10^−1^	4.87 × 10^−2^	Fatty Acid Metabolism	Lipid
Methylmalonate	Urine	0.23	1.61 × 10^−3^	6.49 × 10^−4^	2.67 × 10^−4^	Fatty Acid Metabolism (also BCAA Metabolism)	Lipid
Phenylacetylglycine	Urine	0.16	3.56 × 10^−2^	5.09 × 10^−2^	2.32 × 10^−2^	Acetylated Peptides	Peptide
Fumarate	Urine	−0.22	1.39 × 10^−4^	2.11 × 10^−3^	1.27 × 10^−3^	Krebs cycle	Energy
Citrate	Urine	−0.19	1.40 × 10^−4^	1.89 × 10^−3^	9.58 × 10^−4^	Krebs cycle	Energy
*trans*-Aconitate	Urine	−0.17	1.68 × 10^−2^	1.20 × 10^−1^	5.44 × 10^−2^	Krebs cycle	Energy
Citrulline	Urine	0.18	1.81 × 10^−2^	2.38 × 10^−2^	8.97 × 10^−3^	Urea cycle; Arginine and Proline Metabolism	Amino acid
1-Methylnicotinamide	Urine	−0.11	6.57 × 10^−3^	2.84 × 10^−2^	3.09 × 10^−2^	Nicotinate and Nicotinamide Metabolism	Cofactors and Vitamins

## References

[1] Zhou B., Bentham J., Di Cesare M., Bixby H., Danaei G., Cowan M.J., Paciorek C.J., Singh G., Hajifathalian K., Bennett J.E. (2017). Worldwide trends in blood pressure from 1975 to 2015: A pooled analysis of 1479 population-based measurement studies with 19.1 million participants. Lancet.

[2] Lawes C.M.M., Vander Hoorn S., Rodgers A., Hypertens I.S. (2008). Global burden of blood-pressure-related disease, 2001. Lancet.

[3] Carretero O.A., Oparil S. (2000). Essential hypertension Part I: Definition and etiology. Circulation.

[4] Dietrich S., Floegel A., Troll M., Kuhn T., Rathmann W., Peters A., Sookthai D., von Bergen M., Kaaks R., Adamski J. (2016). Random Survival Forest in practice: A method for modelling complex metabolomics data in time to event analysis. Int. J. Epidemiol..

[5] Posada-Ayala M., Zubiri I., Martin-Lorenzo M., Sanz-Maroto A., Molero D., Gonzalez-Calero L., Fernandez-Fernandez B., de la Cuesta F., Laborde C.M., Barderas M.G. (2014). Identification of a urine metabolomic signature in patients with advanced-stage chronic kidney disease. Kidney Int..

[6] Hocher B., Adamski J. (2017). Metabolomics for clinical use and research in chronic kidney disease. Nat. Rev. Nephrol..

[7] Li J., Zhao F.Q., Wang Y.D., Chen J.R., Tao J.E., Tian G., Wu S.L., Liu W.B., Cui Q.H., Geng B. (2017). Gut microbiota dysbiosis contributes to the development of hypertension. Microbiome.

[8] Wang L.L., Zheng L.Y., Luo R., Zhao X.S., Han Z.H., Wang Y.L., Yang Y.X. (2015). A H-1 NMR-based metabonomic investigation of time-dependent metabolic trajectories in a high salt-induced hypertension rat model. RSC Adv..

[9] Yang M., Yu Z., Deng S., Chen X., Chen L., Guo Z., Zheng H., Chen L., Cai D., Wen B. (2016). A Targeted Metabolomics MRM-MS Study on Identifying Potential Hypertension Biomarkers in Human Plasma and Evaluating Acupuncture Effects. Sci. Rep..

[10] Hiltunen T.P., Rimpela J.M., Mohney R.P., Stirdivant S.M., Kontula K.K. (2017). Effects of four different antihypertensive drugs on plasma metabolomic profiles in patients with essential hypertension. PLoS ONE.

[11] Menni C., Graham D., Kastenmuller G., Alharbi N.H., Alsanosi S.M., McBride M., Mangino M., Titcombe P., Shin S.Y., Psatha M. (2015). Metabolomic identification of a novel pathway of blood pressure regulation involving hexadecanedioate. Hypertension.

[12] Guijas C., Montenegro-Burke J.R., Warth B., Spilker M.E., Siuzdak G. (2018). Metabolomics activity screening for identifying metabolites that modulate phenotype. Nat. Biotechnol..

[13] Quan H., Athirakul K., Wetsel W.C., Torres G.E., Stevens R., Chen Y.T., Coffman T.M., Caron M.G. (2004). Hypertension and impaired glycine handling in mice lacking the orphan transporter XT2. Mol. Cell. Biol..

[14] Jackson A.A., Dunn R.L., Marchand M.C., Langley-Evans S.C. (2002). Increased systolic blood pressure in rats induced by a maternal low-protein diet is reversed by dietary supplementation with glycine. Clin. Sci..

[15] Diaz-Flores M., Cruz M., Duran-Reyes G., Munguia-Miranda C., Loza-Rodriguez H., Pulido-Casas E., Torres-Ramirez N., Gaja-Rodriguez O., Kumate J., Baiza-Gutman L.A. (2013). Oral supplementation with glycine reduces oxidative stress in patients with metabolic syndrome, improving their systolic blood pressure. Can. J. Physiol. Pharmacol..

[16] Bartus M., Lomnicka M., Kostogrys R.B., Kazmierczak P., Watala C., Slominska E.M., Smolenki R.T., Pisulewski P.M., Adamus J., Gebicki J. (2008). 1-Methylnicotinamide (MNA) prevents endothelial dysfunction in hypertriglyceridemic and diabetic rats. Pharmacol. Rep..

[17] Loo R.L., Zou X., Appel L.J., Nicholson J.K., Holmes E. (2018). Characterization of metabolic responses to healthy diets and association with blood pressure: Application to the Optimal Macronutrient Intake Trial for Heart Health (OmniHeart), a randomized controlled study. Am. J. Clin. Nutr..

[18] Holmes E., Loo R.L., Stamler J., Bictash M., Yap I.K., Chan Q., Ebbels T., De Iorio M., Brown I.J., Veselkov K.A. (2008). Human metabolic phenotype diversity and its association with diet and blood pressure. Nature.

[19] Brindle J.T., Nicholson J.K., Schofield P.M., Grainger D.J., Holmes E. (2003). Application of chemometrics to H-1 NMR spectroscopic data to investigate a relationship between human serum metabolic profiles and hypertension. Analyst.

[20] Dietrich S., Floegel A., Weikert C., Pischon T., Boeing H., Drogan D. (2016). Identification of Serum Metabolites Associated with Incident Hypertension in the European Prospective Investigation into Cancer and Nutrition-Potsdam Study. Hypertension.

[21] Ameta K., Gupta A., Kumar S., Sethi R., Kumar D., Mahdi A.A. (2017). Essential hypertension: A filtered serum based metabolomics study. Sci. Rep..

[22] Zaleska M., Olszewski W.L., Durlik M., Miller N.E. (2013). Signaling Proteins Are Represented in Tissue Fluid/Lymph from Soft Tissues of Normal Human Legs at Concentrations Different from Serum. Lymphat. Res. Biol..

[23] Chachaj A., Pula B., Chabowski M., Grzegrzolka J., Szahidewicz-Krupska E., Karczewski M., Janczak D., Dziegiel P., Podhorska-Okolow M., Mazur G. (2018). Role of the Lymphatic System in the Pathogenesis of Hypertension in Humans. Lymphat. Res. Biol..

[24] Machnik A., Neuhofer W., Jantsch J., Dahlmann A., Tammela T., Machura K., Park J.K., Beck F.X., Muller D.N., Derer W. (2009). Macrophages regulate salt-dependent volume and blood pressure by a vascular endothelial growth factor-C-dependent buffering mechanism. Nat. Med..

[25] Machnik A., Dahlmann A., Kopp C., Goss J., Wagner H., van Rooijen N., Eckardt K.U., Muller D.N., Park J.K., Luft F.C. (2010). Mononuclear phagocyte system depletion blocks interstitial tonicity-responsive enhancer binding protein/vascular endothelial growth factor C expression and induces salt-sensitive hypertension in rats. Hypertension.

[26] Hansen K.C., D’Alessandro A., Clement C.C., Santambrogio L. (2015). Lymph formation, composition and circulation: A proteomics perspective. Int. Immunol..

[27] Milasan A., Ledoux J., Martel C. (2015). Lymphatic network in atherosclerosis: The underestimated path. Future Sci. OA.

[28] Olszewski W.L. (2003). The lymphatic system in body homeostasis: Physiological conditions. Lymphat. Res. Biol..

[29] Curigliano G., Lenihan D., Fradley M., Ganatra S., Barac A., Blaes A., Herrmann J., Porter C., Lyon A.R. (2020). Management of cardiac disease in cancer patients throughout oncological treatment: ESMO consensus recommendations. Ann. Oncol..

[30] Lenz E.M., Wilson I.D. (2007). Analytical strategies in metabonomics. J. Proteome Res..

[31] Dona A.C., Jimenez B., Schafer H., Humpfer E., Spraul M., Lewis M.R., Pearce J.T., Holmes E., Lindon J.C., Nicholson J.K. (2014). Precision high-throughput proton NMR spectroscopy of human urine, serum, and plasma for large-scale metabolic phenotyping. Anal. Chem..

[32] Loren C.E., Dahl C.P., Do L., Almaas V.M., Geiran O.R., Morner S., Hellman U. (2019). Low Molecular Mass Myocardial Hyaluronan in Human Hypertrophic Cardiomyopathy. Cells.

[33] Björkblom B., Wibom C., Jonsson P., Mörén L., Andersson U., Johannesen T.B., Langseth H., Antti H., Melin B. (2016). Metabolomic screening of pre-diagnostic serum samples identifies association between α- and γ-tocopherols and glioblastoma risk. Oncotarget.

[34] Xu F., Tavintharan S., Sum C.F., Woon K., Lim S.C., Ong C.N. (2013). Metabolic signature shift in type 2 diabetes mellitus revealed by mass spectrometry-based metabolomics. J. Clin. Endocrinol. Metab..

[35] Chou J., Liu R., Yu J., Liu X., Zhao X., Li Y., Liu L., Sun C. (2018). Fasting serum alphahydroxybutyrate and pyroglutamic acid as important metabolites for detecting isolated post-challenge diabetes based on organic acid profiles. J. Chromatogr. B Analyt. Technol. Biomed. Life Sci..

[36] Wang L., Hou E., Wang L., Wang Y., Yang L., Zheng X., Xie G., Sun Q., Liang M., Tian Z. (2015). Reconstruction and analysis of correlation networks based on GC-MS metabolomics data for young hypertensive men. Anal. Chim. Acta.

[37] Kim M., Jung S., Kim S.Y., Lee S.H., Lee J.H. (2014). Prehypertension-Associated Elevation in Circulating Lysophosphatidlycholines, Lp-PLA(2) Activity, and Oxidative Stress. PLoS ONE.

[38] Phang J.M., Pandhare J., Liu Y. (2008). The metabolism of proline as microenvironmental stress substrate. J. Nutr..

[39] Kaul S., Sharma S.S., Mehta I.K. (2008). Free radical scavenging potential of L-proline: Evidence from in vitro assays. Amino Acids.

[40] Habte-Tsion H.M., Ge X., Liu B., Xie J., Ren M., Zhou Q., Miao L., Pan L., Chen R. (2015). A deficiency or an excess of dietary threonine level affects weight gain, enzyme activity, immune response and immune-related gene expression in juvenile blunt snout bream (*Megalobrama amblycephala*). Fish Shellfish Immunol..

[41] Faure M., Chone F., Mettraux C., Godin J.P., Bechereau F., Vuichoud J., Papet I., Breuille D., Obled C. (2007). Threonine utilization for synthesis of acute phase proteins, intestinal proteins, and mucins is increased during sepsis in rats. J. Nutr..

[42] Fu H., Katsumura Y., Lin M., Muroya Y.F.H., Katsumura Y., Lin M., Muroya Y., Hata K., Fujii K., Yokoya A. (2009). Free radical scavenging and radioprotective effects of carnosine and anserine. Radiat. Phys. Chem..

[43] Zhong Z., Wheeler M.D., Li X., Froh M., Schemmer P., Yin M., Bunzendaul H., Bradford B., Lemasters J.J. (2003). L-Glycine: A novel antiinflammatory, immunomodulatory, and cytoprotective agent. Curr. Opin. Clin. Nutr. Metab. Care.

[44] Tzoulaki I., Iliou A., Mikros E., Elliott P. (2018). An Overview of Metabolic Phenotyping in Blood Pressure Research. Curr. Hypertens. Rep..

[45] Hasegawa S., Ichiyama T., Sonaka I., Ohsaki A., Okada S., Wakiguchi H., Kudo K., Kittaka S., Hara M., Furukawa S. (2012). Cysteine, histidine and glycine exhibit anti-inflammatory effects in human coronary arterial endothelial cells. Clin. Exp. Immunol..

[46] Rodino-Janeiro B.K., Gonzalez-Peteiro M., Ucieda-Somoza R., Gonzalez-Juanatey J.R., Alvarez E. (2010). Glycated albumin, a precursor of advanced glycation end-products, up-regulates NADPH oxidase and enhances oxidative stress in human endothelial cells: Molecular correlate of diabetic vasculopathy. Diabetes Metab. Res. Rev..

[47] Dzieciatkowska M., D’Alessandro A., Moore E.E., Wohlauer M., Banerjee A., Silliman C.C., Hansen K.C. (2014). Lymph Is Not a Plasma Ultrafiltrate: A Proteomic Analysis of Injured Patients. Shock.

[48] Yang J., Villar V.A.M., Jose P.A., Zeng C. (2020). Renal Dopamine Receptors and Oxidative Stress: Role in Hypertension. Antioxid. Redox Signal..

[49] Higashi Y., Maruhashi T., Noma K., Kihara Y. (2014). Oxidative stress and endothelial dysfunction: Clinical evidence and therapeutic implications. Trends Cardiovas. Med..

[50] Matsutomo T., Ushijima M., Kodera Y., Nakamoto M., Takashima M., Morihara N., Tamura K. (2017). Metabolomic study on the antihypertensive effect of S-1-propenylcysteine in spontaneously hypertensive rats using liquid chromatography coupled with quadrupole-Orbitrap mass spectrometry. J. Chromatogr. B Analyt. Technol. Biomed. Life Sci..

[51] Laerke H.N., Mikkelsen L.S., Jorgensen H., Jensen S.K. (2014). Effect of beta-Glucan Supplementation on Acute Postprandial Changes in Fatty Acid Profile of Lymph and Serum in Pigs. Int. J. Mol. Sci..

[52] Rosenson R.S., Brewer H.B., Davidson W.S., Fayad Z.A., Fuster V., Goldstein J., Hellerstein M., Jiang X.C., Phillips M.C., Rader D.J. (2012). Cholesterol efflux and atheroprotection: Advancing the concept of reverse cholesterol transport. Circulation.

[53] Rader D.J., Alexander E.T., Weibel G.L., Billheimer J., Rothblat G.H. (2009). The role of reverse cholesterol transport in animals and humans and relationship to atherosclerosis. J. Lipid Res..

[54] Wang F.H., Liu J., Deng Q.J., Qi Y., Wang M., Wang Y., Zhang X.G., Zhao D. (2019). Association between plasma essential amino acids and atherogenic lipid profile in a Chinese population: A cross-sectional study. Atherosclerosis.

[55] Au A., Cheng K.K., Wei L.K. (2017). Metabolomics, Lipidomics and Pharmacometabolomics of Human Hypertension. Adv. Exp. Med. Biol..

[56] Li P., Wu G.Y. (2018). Roles of dietary glycine, proline, and hydroxyproline in collagen synthesis and animal growth. Amino Acids.

[57] Stakos D.A., Tziakas D.N., Chalikias G.K., Mitrousi K., Tsigalou C., Boudoulas H. (2010). Associations Between Collagen Synthesis and Degradation and Aortic Function in Arterial Hypertension. Am. J. Hypertens..

[58] Melendez-Hevia E., De Paz-Lugo P., Cornish-Bowden A., Cardenas M.L. (2009). A weak link in metabolism: The metabolic capacity for glycine biosynthesis does not satisfy the need for collagen synthesis. J. Biosci..

[59] Kohn J.C., Lampi M.C., Reinhart-King C.A. (2015). Age-related vascular stiffening: Causes and consequences. Front. Genet..

[60] El Hafidi M., Perez I., Banos G. (2006). Is glycine effective against elevated blood pressure?. Curr. Opin. Clin. Nutr. Metab. Care.

[61] Shoulders M.D., Raines R.T. (2009). Collagen structure and stability. Annu. Rev. Biochem..

[62] Tomlinson C., Rafii M., Ball R.O., Pencharz P.B. (2011). Arginine can be synthesized from enteral proline in healthy adult humans. J. Nutr..

[63] Bailie G.R., Uhlig K., Levey A.S. (2005). Clinical practice guidelines in nephrology: Evaluation, classification, and stratification of chronic kidney disease. Pharmacotherapy.

[64] Wannamethee S.G., Shaper A.G., Perry I.J. (1997). Serum creatinine concentration and risk of cardiovascular disease: A possible marker for increased risk of stroke. Stroke.

[65] Sibilitz K.L., Benn M., Nordestgaard B.G. (2014). Creatinine, eGFR and association with myocardial infarction, ischemic heart disease and early death in the general population. Atherosclerosis.

[66] Zhong L., Zhang J.P., Nuermaimaiti A.G., Yunusi K.X. (2014). Study on plasmatic metabolomics of Uygur patients with essential hypertension based on nuclear magnetic resonance technique. Eur. Rev. Med. Pharmacol. Sci..

[67] Kelm M. (2003). The L-arginine-nitric oxide pathway in hypertension. Curr. Hypertens. Rep..

[68] Wittemans L.B.L., Lotta L.A., Oliver-Williams C., Stewart I.D., Surendran P., Karthikeyan S., Day F.R., Koulman A., Imamura F., Zeng L. (2019). Assessing the causal association of glycine with risk of cardio-metabolic diseases. Nat. Commun..

[69] Ding Y., Svingen G.F., Pedersen E.R., Gregory J.F., Ueland P.M., Tell G.S., Nygard O.K. (2015). Plasma Glycine and Risk of Acute Myocardial Infarction in Patients With Suspected Stable Angina Pectoris. J. Am. Heart Assoc..

[70] El Hafidi M., Perez I., Zamora J., Soto V., Carvajal-Sandoval G., Banos G. (2004). Glycine intake decreases plasma free fatty acids, adipose cell size, and blood pressure in sucrose-fed rats. Am. J. Physiol. Regul. Integr. Comp. Physiol..

[71] Newsholme P., Brennan L., Rubi B., Maechler P. (2005). New insights into amino acid metabolism, beta-cell function and diabetes. Clin. Sci..

[72] Tuttle K.R., Milton J.E., Packard D.P., Shuler L.A., Short R.A. (2012). Dietary amino acids and blood pressure: A cohort study of patients with cardiovascular disease. Am. J. Kidney Dis..

[73] Scott D.W., Chen J., Chacko B.K., Traylor J.G., Orr A.W., Patel R.P. (2012). Role of endothelial N-glycan mannose residues in monocyte recruitment during atherogenesis. Arterioscler. Thromb. Vasc. Biol..

[74] Mardinoglu A., Stancakova A., Lotta L.A., Kuusisto J., Boren J., Bluher M., Wareham N.J., Ferrannini E., Groop P.H., Laakso M. (2017). Plasma Mannose Levels Are Associated with Incident Type 2 Diabetes and Cardiovascular Disease. Cell. Metab..

[75] Tzoulaki I., Castagne R., Boulange C.L., Karaman I., Chekmeneva E., Evangelou E., Ebbels T.M.D., Kaluarachchi M.R., Chadeau-Hyam M., Mosen D. (2019). Serum metabolic signatures of coronary and carotid atherosclerosis and subsequent cardiovascular disease. Eur. Heart J..

[76] Yu B., Heiss G., Alexander D., Grams M.E., Boerwinkle E. (2016). Associations Between the Serum Metabolome and All-Cause Mortality Among African Americans in the Atherosclerosis Risk in Communities (ARIC) Study. Am. J. Epidemiol..

[77] Barba I., de Leon G., Martin E., Cuevas A., Aguade S., Candell-Riera J., Barrabes J.A., Garcia-Dorado D. (2008). Nuclear magnetic resonance-based metabolomics predicts exercise-induced ischemia in patients with suspected coronary artery disease. Magn. Reson. Med..

[78] Crawford S.O., Ambrose M.S., Hoogeveen R.C., Brancati F.L., Ballantyne C.M., Young J.H. (2008). Association of lactate with blood pressure before and after rapid weight loss. Am. J. Hypertens..

[79] Menni C., Migaud M., Glastonbury C.A., Beaumont M., Nikolaou A., Small K.S., Brosnan M.J., Mohney R.P., Spector T.D., Valdes A.M. (2016). Metabolomic profiling to dissect the role of visceral fat in cardiometabolic health. Obesity (Silver Spring).

[80] Zheng Y., Yu B., Alexander D., Mosley T.H., Heiss G., Nettleton J.A., Boerwinkle E. (2013). Metabolomics and Incident Hypertension Among Blacks: The Atherosclerosis Risk in Communities Study. Hypertension.

[81] Adnan S., Nelson J.W., Ajami N.J., Venna V.R., Petrosino J.F., Bryan R.M., Durgan D.J. (2017). Alterations in the gut microbiota can elicit hypertension in rats. Physiol. Genom..

[82] Bryniarski K., Biedron R., Jakubowski A., Chlopicki S., Marcinkiewicz J. (2008). Anti-inflammatory effect of 1-methylnicotinamide in contact hypersensitivity to oxazolone in mice; involvement of prostacyclin. Eur. J. Pharmacol..

[83] Chlopicki S., Swies J., Mogielnicki A., Buczko W., Bartus M., Lomnicka M., Adamus J., Gebicki J. (2007). 1-Methylnicotinamide (MNA), a primary metabolite of nicotinamide, exerts anti-thrombotic activity mediated by a cyclooxygenase-2/prostacyclin pathway. Br. J. Pharmacol..

[84] Morris S.M. (2002). Regulation of enzymes of the urea cycle and arginine metabolism. Annu. Rev. Nutr..

[85] Martin-Lorenzo M., Martinez P.J., Baldan-Martin M., Ruiz-Hurtado G., Prado J.C., Segura J., de la Cuesta F., Barderas M.G., Vivanco F., Ruilope L.M. (2017). Citric Acid Metabolism in Resistant Hypertension Underlying Mechanisms and Metabolic Prediction of Treatment Response. Hypertension.

[86] Kang S.M., Park J.C., Shin M.J., Lee H., Oh J., Ryu D.H., Hwang G.S., Chung J.H. (2011). (1)H nuclear magnetic resonance based metabolic urinary profiling of patients with ischemic heart failure. Clin. Biochem..

[87] Akira K., Masu S., Imachi M., Mitome H., Hashimoto T. (2012). A metabonomic study of biochemical changes characteristic of genetically hypertensive rats based on (1)H NMR spectroscopic urinalysis. Hypertens. Res..

[88] Altmaier E., Fobo G., Heier M., Thorand B., Meisinger C., Römisch-Margl W., Waldenberger M., Gieger C., Illig T., Adamski J. (2014). Metabolomics approach reveals effects of antihypertensives and lipid-lowering drugs on the human metabolism. Eur. J. Epidemiol..

